# A systematic review of the caries prevalence among children living in Chernobyl fallout countries

**DOI:** 10.1038/s41598-019-39755-5

**Published:** 2019-03-01

**Authors:** Michael Wolgin, Nicole Filina, Natalia Shakavets, Valentyn Dvornyk, Edward Lynch, Andrej M. Kielbassa

**Affiliations:** 1 Department of Operative Dentistry, Periodontology, and Endodontology, University School of Dental Medicine and Oral Health, Danube Private University (DPU), Steiner Landstraße 124, 3500 Krems, Austria; 2 Department of Pediatric Dentistry, Faculty of Dentistry, Belarusian State Medical University (BSMU), Dzerzhinsky Avenue 83, 220116 Minsk, Belarus; 3 Department of Prosthetic Dentistry and Implantology, Faculty of Dentistry, Ukrainian Medical Stomatological Academy (UMSA), 23 Shevchenko Street, 36011 Poltava, Ukraine; 4 Biomedical and Clinical Research, School of Dental Medicine, University of Nevada (UNLV), 1001 Shadow Lane, Las Vegas, Nevada 89106-4124 United States of America

## Abstract

The present study analyzed the data concerning the caries prevalence in children born and permanently residing in Chernobyl fallout areas. Setting forth to evaluate if differences regarding the caries prevalence can be observed compared to non-contaminated sites of affected East European countries. Methods used to assess the caries prevalence were limited to DMFT/dmft (decayed, missing and filled teeth) for the primary and the permanent dentitions. The databases PubMed, EMBASE/Ovid, Cochrane Library, Scopus, and eLIBRARY were consulted for the electronic literature search. Screening of titles and abstracts followed the MOOSE guidelines, while data extraction and the assessment of the full texts were performed in accordance to the Newcastle Ottawa Scale. The statistical analysis revealed considerable heterogeneity of DMFT/dmft values (from I2 = 94% up to I2 = 99.9%; p < 0.05) in children of different ages (5–7; 12–15; and average of 12 years). Scattering of the weighted mean differences (95% CI) ranged from −1.03 (−1.36; −0.7) to 6.51 (6.11; 6.91). Although individual studies demonstrated a greater prevalence of dental caries in children residing in radiation-contaminated areas, no conclusive statement is possible regarding the effect of small dose radiation on the dentition. Hence, further high-quality epidemiologic investigations are needed.

## Introduction

More than 32 years have passed since the biggest ever radiation accident occurred on April 26, 1986, at the Chernobyl Nuclear Power Plant (CNPP) in the Ukraine. Besides the enormous impact on the CNPP-workers and on more than 50,000 local residents, a huge release of radioactive isotopes caused the evacuation of about 116,000 people from areas surrounding the CNPP, and the subsequent relocation of about 220,000 further inhabitants from the territories of the meanwhile independent countries of Ukraine, Belarus, and Russian Federation^1^. Moreover, the leakage of the radioactive material from the partially destroyed reactor of the CNPP was much greater than previous reactor accidents in Windscale (United Kingdom), Mile Island (USA), and the later accident in Fukushima (Japan) cumulatively together^2^. Furthermore, it has been estimated that the total radioactivity of the material released from the reactor was 200 times greater than the combined release of radioactivity from the atomic bomb explosions in Hiroshima and Nagasaki^3^. Due to both the complicated meteorological situation which persisted immediately after the Chernobyl accident and the long exposure of the destroyed reactor to the atmosphere, radioactive materials were disseminated over a wide area of the Northern Hemisphere^4^. This resulted in a heavy contamination of the territories in Ukraine, Belarus, the European part of the Russian Federation, and, to a lesser extent, of Scandinavia and the rest of Europe^4^. Based on current calculations, about 871,000 km^2^ (which is approximately 11.6% of the total area in Europe) should be contaminated with at least 10–20 Ci/km^2^ of radiocaesium (^134^Cs and ^137^Cs)^1,4,5^. Local zones with high contamination at the level of 1–5 Ci/km^2^ or even higher still exist in parts of Scandinavia, the Alps, Greece, Rumania, Russia, Belarus, and Ukraine itself, thus involving some 211,000 km^2^ (approximately 1.7%) of European ground^4^.

According to an actual report, the vast majority of the five million people residing today in the contaminated areas of Ukraine, Belarus, and Russia currently receive annual effective doses from the Chernobyl fallout of less than 1 mSv^6^, in addition to the natural background doses (worldwide average natural dose to humans is currently about 2.4 mSv per year). However, about 100,000 residents of strongly contaminated areas still receive even significantly more than an additional amount of 1 mSv annually^6^. These circumstances create a specific ecological situation, which may play a modifying role in the development of different human diseases. Considering this aspect, increased attention should be paid to health consequences for children who were born and have been permanently living under this additional exposure to radiation^7,8^. Since the dissemination of radioactive particles, in particular those of radioactive ^137^Cs, largely involves territories of West European countries with a high population density^4,5^, this topic represents a global point of interest for the international community. Moreover, the Fukushima Daiichi nuclear disaster in 2011 has clearly elucidated that nuclear accidents might occur also in the future; therefore, knowledge about the consequences of radioactive contamination for oral health and possible ways to prevent the development of several oral diseases at the contaminated sites will undoubtedly gain significance.

While the negative impact of the low-dose radiation exposure on the general health of children has frequently been described, only little scientific insight has been gathered on the potential influence of small doses of radiation on the development of different oral disorders, such as orofacial and craniofacial malformations, tooth decay, gingivitis, and periodontitis^9^. Several investigations exist concerning the topic of head and neck cancer^10^, as well as of orofacial and craniofacial malformations^11^, but only a few individual studies have been conducted in respect of caries prevalence and caries prevention in children residing in radiation-contaminated areas^9,12–16^. In the light of the respective results, no conclusive statement is possible regarding the effect of small dose radiation on the primary, mixed, or permanent dentitions. Additionally, investigations published in Russian and Ukrainian often cannot be found in traditional electronic databases, or cannot be easily read and interpreted by the English-speaking scientific community due to the lack of familiarity with these Slavic languages. Consequently, this work set forth to systematically review the current literature available concerning the caries prevalence in children residing in radiation-contaminated areas of Ukraine, Belarus, and Russian Federation by multilingual authors and to evaluate if differences regarding the caries prevalence can be observed in these regions compared to non-contaminated sites of affected countries.

## Methods

### Search strategy for identification of studies

In trying to identify the studies to be considered for this review, detailed search strategies were developed for each database to be searched. The search was conducted between February 2017 and February 2018. A systematic research and retrieval of published studies was arranged in accordance with MOOSE (Meta-analyses Of Observational Studies in Epidemiology) guidelines for Meta-Analyses and Systematic Reviews of Observational Studies^17^. Firstly, the databases PubMed, EMBASE/Ovid, The Cochrane Central Register of Controlled Trials, Cochrane Reviews, Scopus and eLIBRARY (a Russian database) were consulted for the electronic literature search. The MeSH terms used in the PubMed search were “Caries OR Caries Prevalence” OR “Caries Intensity” OR “Caries Resistance” OR “Dental Status OR Oral Health” AND “Children” OR “Adolescent” AND “Chernobyl OR Radioactive”. However, these MeSH terms were adopted, broadened and more generalized to “Caries AND Chernobyl” for The Cochrane Central Register of Controlled Trials, Scopus, and Embase databases. In order to perform the electronic literature search in eLIBRARY, the used MeSH terms were translated into Russian. All MeSH terms were finalized by mutual agreement between the first and the senior author of the present study. Moreover, to revise for possible additional papers in English, German, Russian, Ukrainian, and Belarusian the reference lists of identified and relevant studies on the subject were reviewed. In this course, national and international Dissertation Databases were searched for relevant documents. Additionally, successful attempts were made to contact authors and working groups in cases without immediate data availability of certain data necessary to be used in the present systematic review. With this approach five additional investigations could be included in the initial selection. Although no language restrictions were set on the included studies, only six relevant studies were found in languages other than Ukrainian or Russian.

### Eligibility criteria for considering studies for this review

The main aim was to screen for epidemiologic investigations, which included studies that had data collected from Ukrainian, Belarusian, and Russian children with primary, mixed, and permanent dentitions, born and permanently residing in areas with radioactive contamination. The authors framed an answerable and researchable study question to the established PICOT (Population/Patient/Problem, Intervention, Comparison, Outcome, Time) format: “For juvenile patients suffering from caries in primary or/and permanent teeth (Problem/Patient), will an effect of small-dose radiation (Intervention) as compared to an absence of radiation (Control/Comparison) result in a comparable occurrence of tooth decay (Outcome) over time (Time)?” Only observational studies with at least 50 participants per group and studies including a comparison with a non-exposed cohort were used for the present review. To allow for comparison and more conclusiveness on the application and feasibility, the DMFT/dmft index was chosen as the preferred method of testing. Consequently, the primary endpoint was the rate of tooth decay due to dental caries, which comprised the amount of decayed, missing, and filled teeth of the permanent and primary dentition. No secondary endpoints were defined for the present systematic review. Studies of the adult population, studies not reporting outcomes for DMFT/dmft index, case reports, case series without non-exposed cohorts and reviews, as well as studies without possibilities to extract data from presented results were excluded from the main objective, but were considered for explanation of the observed effects.

### Screening process and data collection

**Figure 1:**
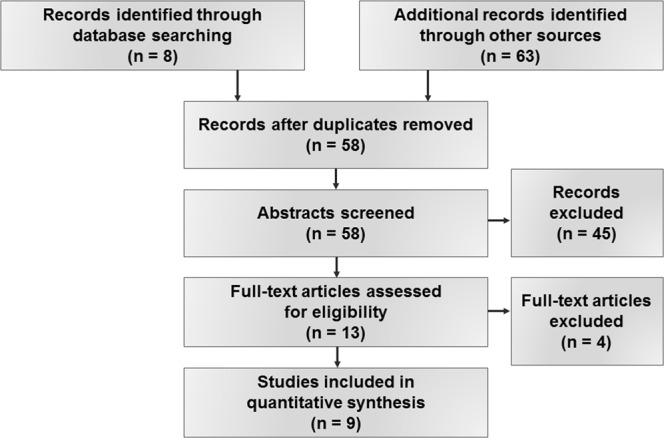
PRISMA Outline.

Two independent reviewers (MW and VD) screened the titles and abstracts of all studies. Obviously irrelevant studies were excluded immediately. Then, both reviewers independently reviewed the remaining full-text articles and selected relevant studies based on the inclusion criteria mentioned above. The same reviewers independently extracted data from each eligible study. Additional information from principal investigators was sought as needed. Any possible dissensions between the authors were eliminated by mutual agreement after discussion. The reviewers were researchers and dentists, each with more than 10 years’ experience in clinical practice and research. Once the abovementioned procedure had been adhered to, nine studies^9,12–16,18–20^ meeting the full inclusion criteria were finally included (Fig. 1). The following details of the studies were extracted: the date and the geographic location of the investigation, degree of on-site radioactive contamination, study type and study method, participants (sample size, type of sample), age of study population and the outcome/measure of reported results. All accepted items were listed to allow for comparison within the studies. Details of these studies are given in Table 1.

**Table 1:** List of characteristics of included studies.

Study	Date	Design	Method	Non-exposed group (NEG)					Exposed group (EG)					Outcome
				Town	Contamination	*n*	Mean Age	DMFT	Town	Contamination	*n*	Mean Age	DMFT	
Spivak (2004)^9^	not specified	cross-sectional	DMFT	Myrhorod (UA)	none	100	13.5	5.7 ± 1.4	Ovruch (UA)	2.69 Ci/km^2^	119	13.5	9.1 ± 3.5	enhanced DMFT values in EG
Sevbitov (2006)^18^	not specified	cross-sectional	DMFT	Sokolniki (RUS)	none	134	13.5	5.76 ± 0.85	Novozybkov (RUS)	15–45 Ci/km^2^	131	13.5	10.21 ± 2.56	enhanced DMFT values in EG
Sevbitov (2014)^16^	not specified	cross-sectional	DMFT	not given	not given	200	20	4.02 ± 0.17	Bryansk (RUS)	>15 Ci/km^2^	400	20	5.94 ± 0.5	enhanced DMFT values in EG
Shapovalova (2001)^15^	not specified	cross-sectional	DMFT	Shishaki (UA)	0.1–0.5 Ci/km^2^	94	7	0.86 ± 0.22	A: Narodichi (UA) B: Ovruch (UA) C: Slavutich (UA)	9.46 Ci/km^2^ Line feed2.69 Ci/km^2^ Line feed2.53 Ci/km^2^	80 52 462	7	A: 6.08 ± 1.25 Line feedB: 7.37 ± 0.79Line feed C: 5.51 ± 0.42	enhanced DMFT values in EG
							12	0.92 ± 0.09				12	A: 4.40 ± 0.05 Line feedB: 4.41 ± 0.49 Line feedC: 2.94 ± 0.24	
							15	3.09 ± 0.39				15	A: 7.25 ± 0.77 Line feedB: not given Line feedC: not given	
Melnichenko (1994)^19^	1987–1990	cross-sectional	DMFT	Rudensk (BY)	none	65	10	5.31 ± 0.21 (1987)	A: Bartolomejevka (BY) Please line feed.B: Bragin (BY) Please line feedC: Wetka (BY)	40 Ci/km^2^ Line feed27 Ci/km^2^Line feed 20 Ci/km^2^	80 343 374	10	A: 4.81 ± 0.20 Line feedB: 5.05 ± 0.15 Line feedC: 5.71 ± 0.17 (1987)	slightly enhanced DMFT values in EG
								4.34 ± 0.21 (1990)					A: 4.88 ± 0.31 Line feedB: 5.24 ± 0.42 C: 5.81 ± 0.32 (1990)	
Melnichenko (1997)^14^	1991–1993	cross-sectional	DMFT	Dzerjinsk (BY)	none	497	7.5	3.86 ± 0.1	Wetka (BY)	20 Ci/km^2^	536	7.5	4.51 ± 0.09	enhanced DMFT values in EG
Smolyar (1995)^20^	1995	cross-sectional	DMFT	Rivne (UA)	none	385	7.5	0.57 ± 0.05 (data only for 6 y. o. children)	Dubrovitsa (UA)	5–40 Ci/km^2^	1089	7.5	2.32 ± 0.24 (data only for 6 y.o. children)	enhanced DMFT values in EG
Petruniv (2012)^13^	not specified	cross-sectional	DMFT	Gorodenka (UA)	none	550	10.5	4.41 ± 0.39	Sniatin (UA)	<1 Ci/km^2^	752	10.5	5.81 ± 0.68	enhanced DMFT values in EG
Kushner (1999)^12^	1998	cross-sectional	DMFT	A: Shklov (BY) Please line feed.B: Ushachi (BY)	<1 Ci/km^2^	105 110	11	A: 4.12 ± 0.38 B: not given	A: Chechersk (BY) B: Bychow (BY) C: Stolin (BY)	15 Ci/km^2^ 5 Ci/km^2^ 5 Ci/km^2^	108 100 102	11	A: 5.18 ± 0.35 B: 3.94 ± 0.58 Line feedC: 4.14 ± 0.25	enhanced DMFT values in EG only in regions with high contamination

### Quality assessment and risk of bias in individual studies

For articles selected in the systematic review, study quality was assessed by means of the customized Newcastle Ottawa Scale (NOS)^21^. Briefly, the assessment was performed in three different domains, such as selection of study groups, comparability of groups and determination of exposure or outcome in dependence from the study type (case-control, cohort, or cross-sectional study) and quality of outcome and adequacy of follow-up, with a maximum score of 9 points. Studies with Newcastle–Ottawa Scale scores of 0 to 3, 4 to 6, and 7 to 9 were rated as having high, moderate, and low risk of bias, respectively.

### Data synthesis and sensitivity analysis

Two authors (MW and NF) conducted the data synthesis and the subsequent data analysis jointly with a representative of the independent Cochrane Center Austria, located in the Department for Evidence-based Medicine and Clinical Epidemiology at the Danube University Krems, Austria. The significance level was set at 5%. Statistical analysis or additional pooling of the collected data was not possible due to the diversity and heterogeneity found in the included studies. For similar reasons, sensitivity analyses were not feasible. Hence, only a narrative synthesis of the presented data was possible.

## Results

### Study selection

The initial electronic search resulted in eight references. Additional records (n = 63) were identified through other sources, such as national and international Dissertation Databases or by the establishment of personal communication with authors and working groups. In the end, 58 studies remained after screening on possible bias of the abstracts and titles. After reading the full text versions and adhering to predetermined inclusion requirements, nine studies remained, while 49 studies were excluded from the subsequent analysis. The most common reasons for the exclusion were:

- use of a deviating index for assessing dental caries prevalence (other than DMFT/dmft);
- studies of the adult population;
- studies without non-exposed cohorts (controls); and/or
- studies not focused to PICO(T) question.

The study selection process is summarized in Fig. 1.

### Quality assessment of included studies

In accordance to the Cochrane Reviewers’ Handbook, the studies were assessed and graded to limit the risk of bias caused by inadequacies in study design, conduct, or analysis. In this case, each study was rated on three different levels according to the Newcastle–Ottawa Scale (NOS), adapted for cross-sectional studies. Of the nine included studies, only one received the score “good quality”^16^, while seven investigations were of “fair” quality^9,12,14,15,19,20^, and one study was considered of “low” quality^13^. The results of the NOS scoring are given in Table 2. Reasons for categorizing the studies as moderate quality were mostly due to inadequate sample sizes, limitations in design (i.e., incorrect measurement of radiation exposure), or inaccuracy in elucidating the obtained data, such as disregarded non-respondent’s data. In addition to the above-mentioned factors, an inappropriate statistical analysis automatically led to a low-quality classification.

**Table 2:** Quality assessment of cross-sectional studies according to the customized Newcastle-Ottawa Scale (NOS).

Study	Assessment of Quality (Newcastle-Ottawa Scale customized for cross-sectional studies)							Total Score	Quality	Risk of Bias
	*Selection*				*Comparability*	*Outcome*				
	Representativeness of the sample	Sample size	Non-respondents	Ascertainment of the exposure (risk factor):	The subjects in different outcome (DMFT) groups are comparable. [Confounding factors controlled.]	Assessment of the outcome	Statistical test			
Spivak (2004)^9^	*	−	−	*	(*), [*] – Levels of fluoride	*	*	******	fair	moderate
Sevbitov (2006)^18^	*	−	−	*	(*), [*] – PI, GI	*	*	******	fair	moderate
Sevbitov (2014)^16^	*	*	−	*	(*), [*] – PI, GI, Resistance to caries	*	*	*******	good	low
Shapovalova (2001)^15^	*	−	−	*	(*), [*] – Chemical composition of enamel	*	*	******	fair	moderate
Melnichenko (1994)^19^	*	−	−	*	(*), [*] – PI, GI	*	*	******	fair	moderate
Melnichenko (1997)^14^	*	−	−	*	(*), [*] – Levels of fluoride, PI, GI	*	*	******	fair	moderate
Smolyar (1995)^20^	*	−	−	*	(*), [*] – Concentration of immunoglobulins	*	*	******	fair	moderate
Petruniv (2012)^13^	−	−	−	*	(*)	*	−	***	poor	high
Kushner (1999)^12^	*	−	−	*	(*), [*] – Concentration of immunoglobulins, PI, GI	*	*	******	fair	moderate

^*^Stars awarded for each quality item according to NOS-based assessment system. - No stars awarded.

### Compilation, characteristics and outcome of included studies

In general, the preferred method of testing (DMFT/dmft) was verified in all selected cross-sectional studies. The outcome of measurements referred to different groups of children, residing either in regions with enhanced radioactive contamination or in regions without any notable contamination. The degree of radioactive contamination with radiocaesium (^137^Cs) was given either in Ci/km^2^ or in kBq/m^2^. To allow for comparison and to reach more conclusiveness, all radiation related measurements were converted into equal physical units (Ci/km^2^). Consequently, the selected studies mentioned four regions among officially designated^22^ different levels of (non-affected, weak, low, and heavy) radioactive contamination in the territories, with less than 1 Ci/km^2^ (<37 kBq/m^2^); 1–5 Ci/km^2^ (37–185 kBq/m^2^), 5–15 Ci/km^2^ (185–555 kBq/m^2^), and 15–40 Ci/km^2^ (555–1480 kBq/m^2^), respectively. Accordingly, six studies reported on populations residing in the areas of heavy contamination^12,14,16,18–20^ while the remaining studies were conducted in areas of low^15^ and weak contamination^9,13^, respectively.

Typically, the study participants were categorized as “exposed” in cases of declared origin and permanent residence in the area of state-approved radioactive contamination. Except for one study^12^, no additional objectification of actually accumulated dose of radiation was conducted. The categorization of “non-exposed” study participants was performed in the same manner. Basically, three different investigation approaches could be detected; either the data was obtained by dental professionals in the framework of mandatory annual preventive dental measures in education centers (dental prophylaxis programs in schools and kindergartens), or during the annual dental check-ups in governmental dental outpatients departments, located in nearby district towns. Alternatively, relevant dental records were obtained by specially formed teams of medical professionals (including dentists), acting as members of national medical care programs, such as “Child victims of Chernobyl”. In all selected papers, except for one study^13^, additional investigations regarding the possible confounding factors, such as water fluoridation, the concentration of immunoglobulins in saliva, hygiene conditions, chemical composition of enamel and its resistance to caries were conducted^9,12,14–16,18–20^. However, due to the strong diversity of confounding factors and obvious methodical differences, no summarizing of these data was possible. Nevertheless, most studies allowed for data extraction in respect of different age categories. Thus, four of nine studies^13–15,20^ reported on the DMFT/dmft measurements of early school-age children (5–7 years old), while six studies^9,12–15,18^ covered the aspects of caries prevalence in adolescents (12–15 years old). Indeed, one study did not permit any age distribution^16^; however, three of eight age-dependent studies^13,14,19^ provided mean values for the entire study population, thus enabling the assessment of summarized results. In the following, the principal results are presented in relation to the age and the degree of radioactive contamination.

### Early school-age children (5–7 years old)

**Figure 2:**
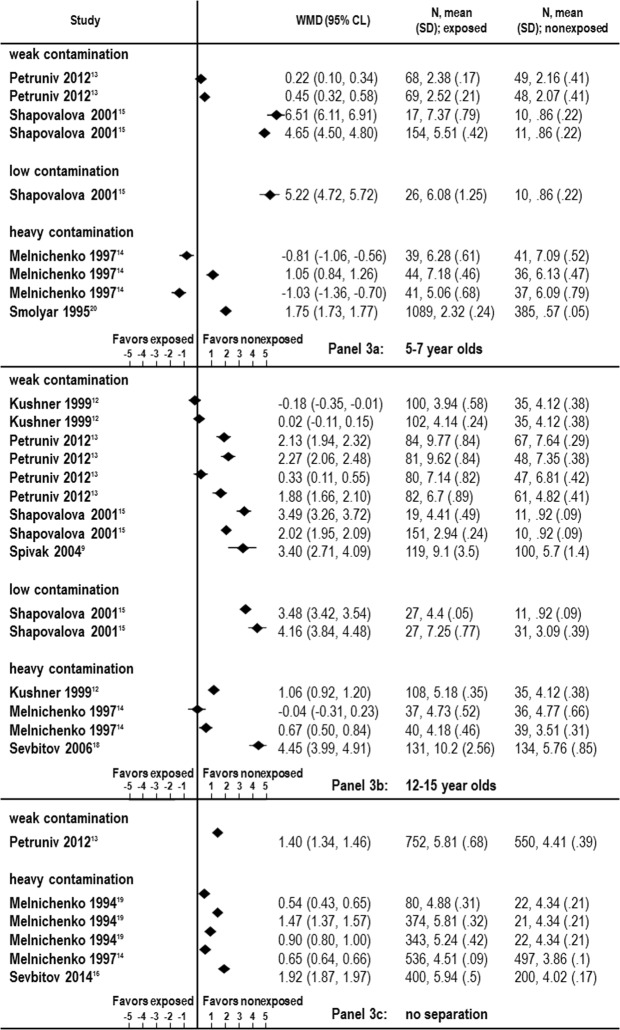
Weighted Mean Difference (WMD), sample size (n), standard deviation (SD) for selected studies in three different age groups; 2a (5–7 year olds), 2b (12–15 year olds) and without age separation (2c).

The data for this age category were available from four selected studies^13–15,20^. Three studies were published in Ukraine in 1995^20^, 2001^15^, and 2012^13^, while the remaining investigation came from Belarus, and was published in the year 1997^21^. In one study the data were only obtained from an area with a weak level of radioactive contamination^13^, while one study was conducted with the population living in three different towns with both weak and low contaminations^15^, and two additional studies collected data exclusively from four heavily contaminated towns^14,20^. The entire number of cases in all regions amounted to 1,547 exposed children vs. 627 unexposed controls. Although the total number of cases might seem large at a first glance, only one study^20^ showed an appropriate sample size (1089 exposed vs. 385 unexposed cases). This study demonstrated a Weighted Mean Difference (WMD) of 1.75 with the 95% CI (confidence interval) of 1.73, 1.77. In contrast, another study^14^ demonstrated even negative values of WMD (95% CI) [−0.81 (−1.06, −0.56) and −1.03 (−1.36, 0.70)], thus leading to the assumption that radiation should have reduced the prevalence of dental caries in this population. The data for both studies were collected in children living in four towns with heavy radioactive contamination. All remaining^13,15^ studies were characterized by inappropriate sample sizes (ranging from n = 17 for exposed, and n = 10 for unexposed to n = 154 for exposed and n = 49 for unexposed patients). Generally speaking, the statistical analysis of data from these four relevant studies revealed a considerable heterogeneity of DMFT/dmft values (from I^2^ = 99.6% to I^2^ = 99.9%; p < 0.05) in children of the studied age category. Therefore, a sufficient pooling of data was not possible. The results for this age category are summarized in Fig. 2a.

### Adolescents (12–15 years old)

The data for this age category were available from six selected studies^9,12–15,18^. Two studies^13,15^ were published in Ukraine in 2001 and 2012, while two further Belarusian studies^12,14^ dated from 1997 and 1999; one study^18^ was published in Russia in 2006, while the remaining investigation^9^ came from the US in 2004; it should, however, be mentioned that the data for the latter study were collected in two Ukrainian towns in the year 2003.

In two of six studies, the data were obtained solely from areas with a weak level of radioactive contamination^9,13^. One study was conducted in a population living in four different towns with weak and low contamination^15^. One study reported on data from two different towns with both weak and heavy contamination^12^, while two remaining studies collected data from three different heavy contaminated towns only^14,18^. The entire number of cases for this age category in all regions amounted to 1,188 exposed children vs. 700 unexposed controls. Although the distribution of cases and controls among all investigations in this age category was more homogenous if compared with the corresponding records of the already mentioned studies in the age category “early school-age children”, none of these studies was equipped with an appropriate sample size. The study with the biggest sample size^16^ for cases and controls (n_case_ = 131 and n_contro l_ = 134, respectively) demonstrated a Weighted Mean Difference (WMD) of 4.45 with the 95% CI (confidence interval) of 3.99 to 4.91. In contrast, another study^14^ demonstrated again negative values of WMD (95% CI) [−0.04 (−0.31, 0.23)], thus disproving any effect of radiation. Comparable with the situation in the age category “early school-age children”, the data for these two contradictory studies^14,18^ were also collected in children living in four towns with heavy radioactive contamination.

The same pattern could be recognized in studies conducted in regions with weak radioactive contamination^12,13^. While studies with the largest and the second largest sample sizes^9,15^ demonstrated positive WMD (95% CI) [2.02 (1.95, 2.09) and 3.40 (2.71, 4.09), respectively], another study^12^ again revealed negative corresponding values of −0.18 (−0.35, −0.01). All remaining studies were again characterized by inappropriate sample sizes (ranging from n = 19 for exposed and n = 10 for unexposed to n = 108 for exposed and n = 31 for unexposed participants). The statistical analysis of data from these six relevant studies again revealed considerable heterogeneity of DMFT/dmft values (from I^2^ = 94.0% to I^2^ = 99.7%; p < 0.05) in children of the studied age category. Therefore, a sufficient pooling of data was also not possible in the group of adolescents; the corresponding results for this age category are shown in Fig. 2b.

### Age groups summarized

In this category, the results were available from four studies; among them, there are two already mentioned investigations^13,14^, which revealed collected data either in category “early school-age children” or in “adolescents”, and presented additionally the mean data for all age groups. Two further studies^16,19^ included data without any separation of ages, thus, the average age in these four investigations was about 12 years. One study was published in Ukraine^13^ in 2012 and another one emerged from Russia^16^ in 2014, while the two remaining investigations came from Belarus^14,19^, and were published in 1994 and 1997 by the same working group. The Ukrainian study^13^ enclosed data from the area of weak radioactive contamination, while three further investigations^14,16,19^ were conducted with the populations living in five different heavily contaminated towns (one in Russia and four in Belarus). The entire number of cases in all regions amounted to 2,485 exposed children vs. 1,312 unexposed controls. With three (out of six) heavily contaminated sites no effect of radiation for DMFT/dmft was observed, while three remaining investigations reported enhanced DMFT/dmft-values even in regions with weak contamination. Overall, the evidence for a significant heterogeneity of data, collected in this age category was again clearly noticeable. Thus, the scattering of WMD (95% CI) ranged between 0.54 (0.43, 0.65) and 1.92 (1.87, 1.97), and the statistically determined heterogeneity amounted to I^2^ = 99.8% (p < 0.05). The results for this age category can be obtained from Fig. 2c.

## Discussion

The present work aimed to review the currently available literature concerning the caries prevalence in children residing in radiation-contaminated areas of Ukraine, Belarus, and the Russian Federation after the Chernobyl nuclear disaster in 1986. In this process, the data of nine studies (out of 58 published papers) were considered for analysis. Overall, it was somewhat discouraging to realize the limited amount of studies matching the inclusion criteria. Although the initial insight into selected studies raised no serious doubts concerning the methods of testing, sample size, or the composition of target groups, a closer and detailed inspection of the selected studies revealed several problems; in particular, low methodological quality, diversity and heterogeneity was a concern. Indeed, the present investigation was initially designed as a meta-analysis; however, after failing to obtain homogeneous data, we decided to transform our data into a systematic review (which was in accordance with a recommendation of the Cochrane Center Austria, Krems, Austria). Subsequently, the main components of the reviewed studies should enjoy a prominent place in the following discussion.

In general, the studies showed major concerns regarding selection bias, and this particularly was related to sample size and lack of information about non-responders. Thus, in two Ukrainian studies the number of investigated adolescents was at least twice as high as the number of early school-age children^13,15^, while the composition of the control (non-exposed) groups in these studies was uniform. Consequently, both authors concluded that the caries prevalence in children residing in radiation-contaminated areas of Ukraine was significantly increased if compared with non-contaminated regions. Since the prevalence and severity of dental caries among adolescents are in general higher than in the groups of early school-age children^23^, the aforementioned facts could represent a major design flaw. Interestingly enough, a study from Belarus^14^ provided contradictory results, while the composition of exposed cohorts corresponded accordingly (124 exposed early school-age children and 77 exposed adolescents) with Ukrainian papers. Hence, it should be stressed that the common representativeness of such studies does not match the requirements^24^ of the STROBE statement (Strengthening The Reporting of Observational Studies in Epidemiology).

In contrast, the representativeness of samples and the ascertainment of exposure would appear to be less of a problem. On the one hand, almost all exposed subjects lived in areas with at least 2.53 Ci/km^2^ of radioactive contamination (only one paper^13^ described subjects residing in areas with radioactive contamination less than 1 Ci/km^2^ as the exposed cohort). On the other hand, the authors of one Ukrainian study included children from the towns with 0.1–0.5 Ci/km^2^ into the non-exposed group^15^, while all other authors paid attention to choose participants from totally clean areas. The size of the localities (exposed and non-exposed) and the number of inhabitants in these towns were reasonably comparable within the individual studies except for one study^20^ comparing the non-contaminated medium-sized town Rivne (approximately 250,000 inhabitants) to the radioactively contaminated small town Dubrovitsa (approximately 10,000 people). Additionally, it should be mentioned that the ascertainment of personal exposure to radioactivity was performed by means of electron spin resonance (ESR) spectroscopy of tooth enamel only in one investigation^12^. In the remaining papers,^Refs^ no measurements of personal radioactive exposure have been described; this obviously was based on the assumption that the individuals who are permanently living in the officially recognized areas with radioactive contamination should have been exposed. This is, however, not always the case, because the radioactive “hot spots” are unequally distributed even in the so called Chernobyl Exclusion Zone^4,5^. Therefore, it is certainly feasible that there could be considerable differences between different study participants concerning their actual level of absorbed dose. From this perspective, the ascertainment of personal exposure to radiation should rather be a mandatory diagnostic prerequisite prior to initiation of any further investigation^25–28^. Moreover, only four studies provided the information concerning the year of the investigation^12,14,19,20^, while in the remaining five investigations the time of data collection was not additionally specified^9,13,15,16,19^. On the one hand, this fact can be considered as design dependent bias; on the other hand, with respect to the long physical half-life of respective radionuclide (approx. 30 years for ^134^Cs and ^137^Cs), it is conceivable to talk about sustainable effects of radiation through the last 30 years.

When discussing the comparability of individual studies, it should be emphasized, that the authors of the present review have reasonable doubts (at least from today’s point of view) concerning the validity of DMFT as a sole preferred method of testing. Nowadays, the combination of DMFT index with ICDAS-II seems to be the most promising method of testing, which can provide more information (in particular within the D [decayed]-component) about the stage, activity and extent of dental caries^29^. Thus, the utilization of DMFT in selected studies might have resulted in low inter-rater reliability, whereby the median values of DMFT varied between 0.57 ± 0.05 and 7.09 ± 0.52 in cohorts of non-exposed and from 2.32 ± 0.24 to 7.18 ± 0.46 in cohorts of exposed early school-age children.

A similar trend was clearly noticeable also in other age groups. Notwithstanding, the importance of careful recording of such sensitive data, the testing procedure has only scarcely been described in any of the selected studies. Therefore, it is difficult to recognize how many raters were involved in this process, if different raters were involved, how these assessors were calibrated, and if additional information (e. g. proximal caries) could be obtained by means of intraoral radiographs. Only an American study provided a conclusive description of the testing procedure^9^, according to which two non-calibrated dentists performed their clinical examinations in two different Ukrainian towns (Ovruch, 2.69 Ci/km^2^; and Myrhorod, non-exposed)^9^. Referring to this description, the expectation of any form of appropriate inter-rater reliability should clearly be scrutinized.

Unfortunately, there are, however, some more inconsistencies in that latter study^9^. Most conspicuous was the sustainable shift of DMFT values in favor of M-components (missing teeth), indicating an unusually high number of tooth extractions due to caries reasons in adolescents (13–14 year olds), regardless of the contamination level (mean ± SD; 4.4 ± 0.9 for exposed vs. 4.3 ± 0.6 for non-exposed children). In contrast, comparable investigations, which have also been included in the present analysis, displayed much lower M-components in this age group, with maximum values of 1.29 ± 0.16 for exposed and 1.21 ± 0.12 for non-exposed children^13^. These discrepancies would seem astonishing, but could, potentially, be easily explained. Since two local dentists collected the data for the abovementioned study^9^ in Ukraine, they most probably have used the Ukrainian index КПВ (К - Карієс [decayed], П – Пломбовано [filled], B – Видалено [missing]) or the Russian index КПУ (К – кариозные [decayed], П – пломбированные [filled], У – удалённые [missing]), which are both equivalent to DMFT. In the English language publication, however, the traditional DMFT was applied. On closer examination, it would seem striking that the position of capitals M (missing teeth in DMFT) and B (missing teeth in КПВ) is transposed, whereby the capital B corresponds rather to the F component (filled teeth in DMFT). If one accepts that all M-values in this investigation actually belong to the F-component, the result would be in line with all other investigations conducted in this field. However, these thoughts are speculative in nature, since the corresponding authors of the respective paper^9^ did not respond to our repeated queries regarding the possible drawback described above.

Interestingly, the number of decayed teeth seems to be higher in children and adolescents residing in regions with radioactive contamination than in children of non-exposed towns^9,13,18^. Concurrently, the number of filled teeth is substantially comparable in both cohorts^9,13^ and sometimes even tends to be lower in exposed children, if compared with the non-exposed ones^18^. These observations encourage suspicions about the general lack or growing deterioration of dental care in radioactive contaminated regions.

To a certain extent, the information about the state of dental care in affected towns can be obtained from the selected studies. While most authors^9,15,16,18,20^ clearly attribute the increased rates of caries directly or indirectly to radiation effects, a Belarusian author made the under-staffed medical and dental personnel in radioactively polluted areas a subject of discussion^19^. According to the Public Health Ministry of the Republic of Belarus^30^, in the year of 2010 the total number of dentists in the region of Homel (some 1,500,000 inhabitants), which was the most affected area from radioactive fallout after the Chernobyl accident, amounted to only 328 employees. Therefore, the dentist supply rate for the population in this region amounted to 2.3 dentists per 10,000 people. It should be added that about a third of this population lives in the city of Homel (some 500,000 inhabitants), where probably the majority of the dentists is concentrated. In contrast, the non-contaminated region around Minsk with a similar population (the 2 million city of Minsk is excluded) had, at the same time, 534 dentists, and this supply rate was considered nearly twice as high if compared to the region of Homel. Taking these facts into account, the sole role of radiation must be seriously weighed against other options. Additionally, it might be relevant to compare DMFT/dmft-values in children that have inhabited the affected regions shortly before the Chernobyl disaster with the actual data. However, the search for suitable sources, concerning the caries prevalence prior to nuclear accident was, however, not successful. It must be emphasized that during the Soviet period, the remote region of Polesie was rather insignificant for epidemiologic data acquisition, due to various reasons such as low population density and the secrecy of this region due to the presence of restricted military areas.

Nonetheless, the potential impact of radiation on oral health cannot be completely denied. On the contrary, the analyzed studies contain interesting attempts of an explanation for possible mechanisms, which can promote the development of carious lesions. Of particular interest might be the altered composition of saliva. Thus, analyzed studies reported decreased concentrations of SIgA, IgG and IgM in saliva^20^, reductions of salivary flow rates, and slow degradation of minor salivary glands in children and adolescents residing in radioactive contaminated areas^19^. Another explanation may be different changes in the chemical composition of dental enamel^15^ and the related reduced acid-resistance^14^, which have also been described. Admittedly, all these facts still need to be confirmed by at least three well-designed independent epidemiological studies with appropriate sample size and under consideration of all possible confounders.

Finally, is worth mentioning that the impact of small dose radiation described above do not appear comparable with the consequences of radiotherapy for oral health. The cumulative exposure levels during the radiotherapy are substantial higher (approx. 60 Gy)^31^ than the expected exposure due to soil contamination with radiocaesium. Studies have shown that the absorbed dose rate (even in the year of 1986) reached only 1.3–6.0 Gy h(−1) in the central areas of the Chernobyl Exclusion Zone. In 1988 and 1990, the total absorbed dose rates were 1.3 and 0.42 Gy h(−1), respectively. In 1995, 2000, and 2005, the total absorbed dose rates rarely exceeded 0.00023, 0.00018, and 0.00015 Gy h(−1), respectively^32^. Finally, previous studies have clearly shown, that therapeutically irradiated dentine and enamel are not more susceptible to de- or remineralization than non-irradiated, if adequate oral hygiene techniques are implemented^33–35^. Thus, it would seem reasonable to deduce that dental hard substances should not be affected by small dose radiation; instead, organic tissues (i.e., salivary glands) might be more prone to radiation damage^31^, thus indicating a possible focus of future interest.

## Conclusions

Summarizing the currently available studies, conducted between 1987 and 2014, and concerning the question of caries prevalence in children residing in radiation-contaminated areas of Eastern Europe, the multilingual authors of the present investigation could not demonstrate any relationship between the caries prevalence and the degree of radioactive contamination. Moreover, any obvious differences between the caries prevalence in children of contaminated and non-contaminated regions in Ukraine, Belarus, and the Russian Federation could not be revealed. Notwithstanding, the absence of a sound epidemiological evidence for such a specific ecologic situation, which might play a modifying role in the development of dental caries, should justify and encourage the interest in planning and conducting more high-quality studies.

## Data Availability

The datasets generated and/or analyzed during the current study are available from the corresponding author on reasonable request.
