# Publisher Correction: A systematic review of the caries prevalence among children living in Chernobyl fallout countries

**DOI:** 10.1038/s41598-020-67583-5

**Published:** 2020-06-22

**Authors:** Michael Wolgin, Nicole Filina, Natalia Shakavets, Valentyn Dvornyk, Edward Lynch, Andrej M. Kielbassa

**Affiliations:** 1 Department of Operative Dentistry, Periodontology, and Endodontology, University School of Dental Medicine and Oral Health, Danube Private University (DPU), Steiner Landstraße 124, 3500 Krems, Austria; 2 Department of Pediatric Dentistry, Faculty of Dentistry, Belarusian State Medical University (BSMU), Dzerzhinsky Avenue 83, 220116 Minsk, Belarus; 3 Department of Prosthetic Dentistry and Implantology, Faculty of Dentistry, Ukrainian Medical Stomatological Academy (UMSA), 23 Shevchenko Street, Poltava, 36011 Ukraine; 4 Biomedical and Clinical Research, School of Dental Medicine, University of Nevada (UNLV), 1001 Shadow Lane, Las Vegas, NV 89106-4124 USA

Correction to: *Scientific Reports* 10.1038/s41598-019-39755-5, published online 01 March 2019

This original version of this Article contained errors in Table 1. Due to a production error, the version of Table 1 originally published with this Article contained typesetting information. This has now been corrected in the PDF and HTML versions of the paper.

